# HLA Alleles Association with Changes in Bone Mineral Density in HIV-1-Infected Adults Changing Treatment to Tenofovir-Emtricitabine or Abacavir-Lamivudine

**DOI:** 10.1371/journal.pone.0093333

**Published:** 2014-03-28

**Authors:** Hila Haskelberg, Damien V. Cordery, Janaki Amin, Anthony D. Kelleher, David A. Cooper, Sean Emery, on behalf of the STEAL Study Group

**Affiliations:** 1 The Kirby Institute, University of New South Wales, Sydney, Australia; 2 St Vincent's Centre for Applied Medical Research, Darlinghurst, New South Wales, Australia; Imperial College London, United Kingdom

## Abstract

**Background:**

There are limited data regarding the influence of human leukocyte antigen (HLA) polymorphisms on reduced bone mineral density (BMD). We investigated the relationship between HLA supertypes and BMD in HIV-infected adults changing their existing treatment to tenofovir-emtricitabine (TDF-FTC) or abacavir-lamivudine (ABC-3TC) in the STEAL study.

**Methods:**

Lumbar spine and right hip BMD were measured by Dual-energy X-ray absorptiometry (DXA). HLA genotypes at the 2-digit level were classified into class I and II supertypes. Student's t-tests were used to test the association between HLA supertypes and changes in hip and spine BMD over 96 weeks for the whole cohort and stratified by randomised groups. The relationship between HLA supertypes and BMD was also assessed in the subgroup of participants that were naïve to both ABC and TDF at study entry.

**Results:**

Class II supertypes were mainly associated with hip BMD change. Overall, compared to participants not carrying HLA-DQ3, participants expressing DQ3 had less bone loss over 96 weeks at both the hip and spine (hip: 0.003 vs. −0.006 g/cm^2^, 95%CI 0.002 to 0.017, p = 0.016; spine: 0.006 vs. −0.006 g/cm^2^, 95%CI 0.001 to 0.023, p = 0.041). In participants that were naïve to both ABC and TDF at baseline and randomised to TDF-FTC, DQ3 was significantly associated with less bone loss compared with those not carrying DQ3 (hip: 0.001 vs. −0.032 g/cm^2^; diff 0.033; 95%CI 0.017 to 0.049; p<0.001; spine: 0.007 vs. −0.023 g/cm^2^; diff 0.035; 95%CI 0.014 to 0.056; p = 0.001).

**Conclusions:**

In this cohort of HIV-infected adults, there was an association between bone status and HLA supertypes, particularly HLA-DQ3.

**Trial Registration:**

Clinicaltrials.gov NCT00192634

## Introduction

The major histocompatibility complex (MHC) in humans, known as human leukocyte antigen (HLA) region, is a highly polymorphic genetic system that encodes cell-surface antigen-presenting proteins. It plays an important role in host immune function and is associated with several infectious and inflammatory diseases. The skeletal and immune systems are closely related through various cellular and molecular and interactions [1]. The strong association between disorders of the immune system and susceptibility to osteoporosis [2], [3] may indicate that there are shared genetic factors as well.

BMD has a high heritability: twin and family studies show that between 50 and 85% of the variance in peak BMD is genetically determined [4], [5]. Several genomic regions have been identified that influence BMD, particularly the Receptor activator of nuclear factor kappa-B (RANK) ligand, RANK, and osteoprotegerin gene pathways [6]. In a study of young Japanese women, the HLA-A*24-B*07-DRB1*01 haplotype was strongly associated with a lower peak bone mass within the normal range [7]. Osteoporosis has a strong genetic component [8], yet there are limited data regarding the influence of HLA polymorphisms on reduced bone mineral density (BMD) and osteoporosis. A study in postmenopausal women showed significant association between HLA alleles and bone loss, with increased frequency of HLA-DR15 (*P* = .019) and -DQ6 (P = .026) supertypes in osteopenic and osteoporotic women compared with healthy controls [9]. HLA-B27 transgenic rats show increased levels of bone resorption markers and reduced BMD [10].

Variation within the HLA genes, specifically HLA-B alleles, has been associated with HIV disease progression: HLA-B35 was linked with rapid progression to AIDS [11], while HLA-B27 and B57 are associated with slower HIV disease progression [12]. Several studies identified associations between HLA and complications of specific antiretroviral drugs. Genetic screening in HIV-infected patients for HLA-B*5701 is used to predict abacavir hypersensitivity [13], and HLA-B*3505 has been associated with nevirapine-induced rash [14]. Recently, HLA-B*4001 was suggested as a genetic risk factor for stavudine-associated lipodystrophy in Thai patients [15], though this association was not found in Caucasians patients [16]. Cordery et al. showed that HIV-infected adults carrying HLA-A01, B08 or DQ2 supertypes may be resistant to peripheral fat-loss induced by thymidine analogue nucleotide reverse transcriptase inhibitors (NRTIs) [17]. Genetic analysis of bone loss in the setting of HIV-infection and treatment has not been studied.

In the STEAL study, patients randomised to coformulated tenofovir/emtricitabine (TDF-FTC) had a significant BMD decrease at the spine (−0.005 vs. 0.008 g/cm^2^, p = 0.017) and the hip (−0.007 vs. 0.004 g/cm^2^, p = 0.006) compared with those who were randomised to abacavir/lamivudine (ABC-3TC) over 96 weeks. Randomisation to TDF-FTC was an independent risk factor for bone loss [18]. The aim of this analysis was to explore the associations between specific HLA supertypes and bone mineral density (BMD) in HIV-infected adults. We then aimed to examine the association between HLA supertypes and BMD change over 96 weeks and whether these relationships were affected by treatment change to TDF-FTC or ABC-3TC.

## Methods

### Study design

STEAL was an open-label, prospective, randomised, non-inferiority study that compared simplification of current NRTIs to fixed-dose combination TDF-FTC or ABC-3TC over 96 weeks in 357 adults with plasma HIV viral load <50 RNA copies/ml [19]. Participants were recruited from 30 Australian sites. HLA-B*5701 positivity was part of the exclusion criteria for the main study [19]. The supporting CONSORT checklist and STEAL protocol are available as supporting information; see Checklist S1, and Protocol S1.

### Ethics Statement

The study was approved by each site's Human Research and Ethics Committee and registered at Clinicaltrials.gov (NCT00192634). The specific ethics committees that gave approval for the STEAL study are: St Vincent's Hospital Human Research Ethics Committee (HREC), South Eastern Sydney/Illawarra Area Health Service HREC, Harbour HREC of Northern Sydney Central Coast Health, North Coast Area Health Service HREC, Sydney West Area Health Service HREC, Sydney South West Area Health Service HREC, Alfred Hospital EC, Southern Health HREC, Melbourne Health HREC, The Prince Charles Hospital HREC, Cairns & Hinterland Health Service District EC, Gold Coast Health Service District HREC, Royal Brisbane and Women's Hospital HREC, Flinders Clinical Research EC, Royal Adelaide Hospital Research EC, Royal Perth Hospital EC. Each participant signed a written informed consent before enrolment.

### Bone Mineral Density

Dual-energy X-ray absorptiometry (DXA) of the lumbar spine and right hip were performed for each participant at the same imaging facility on the same bone densitometer, at baseline, week 48, and week 96, using a standardized protocol. BMD scans were not centrally analysed. DXA instruments varied between sites (GE-Lunar in 72% of sites); randomisation was stratified by site, and therefore by model of DXA scanner.

### HLA genotypes

The genotype of five HLA genes (HLA-A, HLA-B, HLA-DP, HLA-DQ and HLA-DR) was determined by the Australian Red Cross Blood Service, using sequence-based typing of DNA amplified from baseline blood samples. HLA genotypes were predominantly available at the 2-digit level. The coding of genotypes into supertype was described in detail elsewhere [17]. In short, HLA class I alleles were classified into supertypes according to the associations defined by Sidney et al. [20] and HLA class II alleles according to Doytchinova and Flower [21]. Two-digit alleles that belong to two or more supertype groups were assigned to the supertype that exclusively contained the most common alleles for that 2-digit genotype. Alleles that were shared between supertypes remained unclassified.

### Statistical analyses

All analyses were performed on the per protocol data, defined as data collected on participants while on randomised strategy according to the STEAL protocol. A cross-sectional analysis of the association between HLA supertypes and hip and spine BMD at baseline was carried out using Student's t-tests. In an exploratory analysis, the interaction between HLA and type of NRTI at study entry (TDF vs. non-TDF) on baseline hip and spine BMD was tested using linear regression for the interaction term. The relationship between the change in hip and spine BMD from baseline to week 96 was tested using Student's t-test as well. Analyses were carried out for each HLA supertype stratum within randomised treatment group. The following supertypes were tested: HLA-A01,-02-03 and −24; HLA-B07, −08,−27,−44, −58 and −62; HLA-DPw1, −w2, −w4, and −w6, HLA-DQ1, −2 and −3; and HLA-DR1, −3, −4, −5, and −9. Groups with n<10 were not included. There was no correction for multiple comparisons. A pre-defined sensitivity analysis to test the relationship between HLA and change in BMD over 96 weeks was carried out for participants that were naïve to ABC and TDF at baseline. Interaction between HLA supertypes randomized arm in predicting change in BMD was tested using linear regression for the interaction term.

Statistical significance was defined as a 2-sided α of 0.05 (for exploratory analyses p<0.1 was included). Statistical analyses were performed with STATA, version 12.0 (Statacorp)

## Results

**Figure 1:**
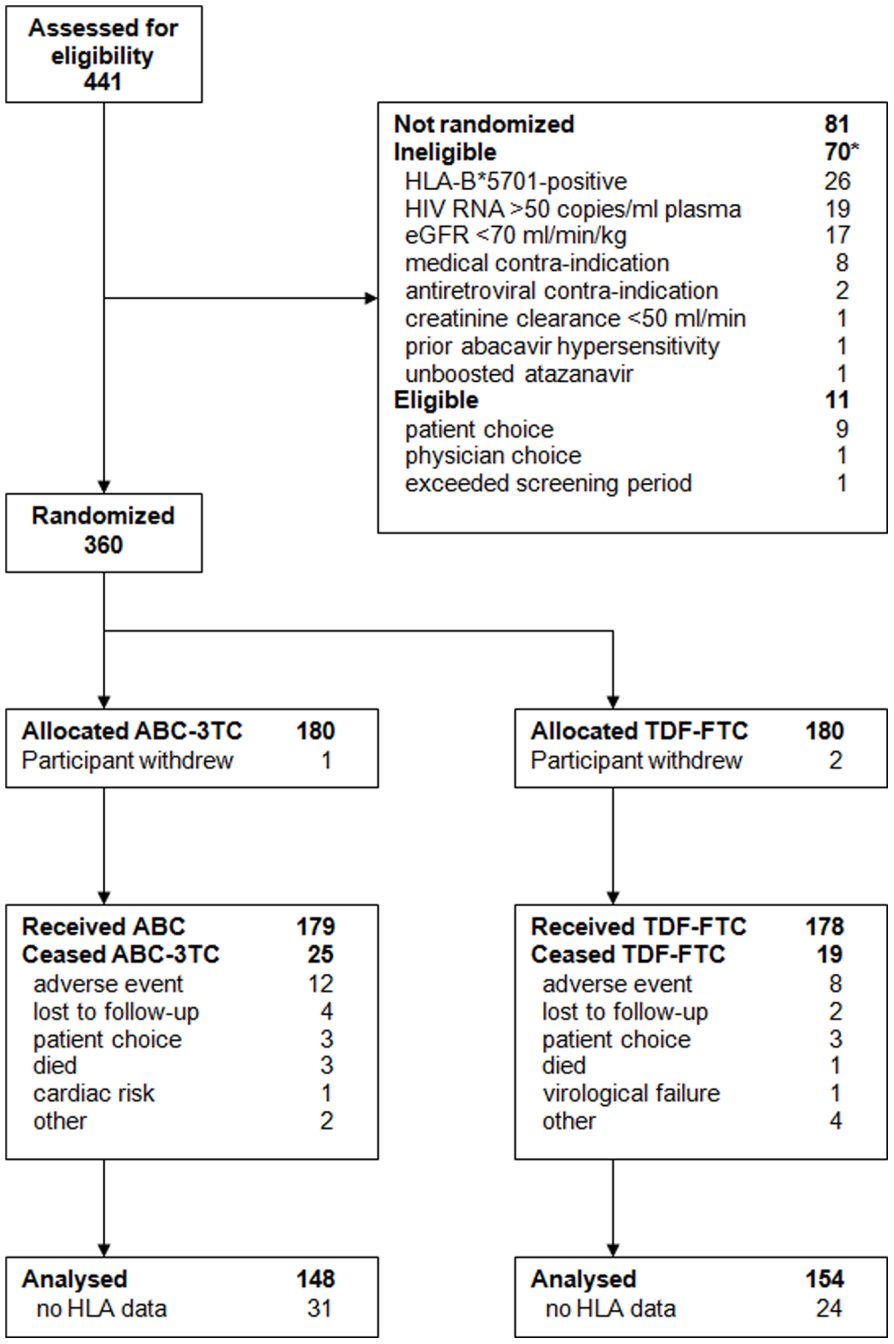
Patient disposition. * Some patients were ineligible for more than one reason.

Patient disposition is outlined in Figure 1. Of 357 participants enrolled in the parent STEAL study, 302 had available HLA genotyping and DXA data at baseline (85% of main study population). Baseline characteristics of the population analysed were similar to main study [19] and well balanced between arms (Table 1).

**Table 1:** Baseline characteristics.

Baseline characteristics	ABC-3TC (n = 148)	TDF-FTC (n = 154)	Main Study (n = 357)
Age (years)	46.1±8.9	44.6±8.3	45.1±8.6
Male (n, %)	146 (99)	150 (97)	349 (98)
Ethnicity (n, %)			
White	126 (85)	133 (86)	307 (86)
Asian	11 (7)	9 (6)	25 (7)
Other	11 (8)	12 (8)	25 (7)
HIV duration (min, max; years)	10.2 (1, 23)	10.5 (0.9, 26)	10.3 (0.9, 26)
CD4+ count	623±316	601±260	612±282
NRTI exposure			
Prior ABC (n, %)	26 (18)	29 (19)	73 (20)
Prior TDF (n, %)	47 (32)	50 (32)	107 (30)
NRTI duration (years)	5.7±3.4	5.9±3.7	5.8±3.6
Current protease inhibitor (n, %)	35 (24)	33 (21)	83 (23)
Anthropometric factors			
Weight (kg)	76.6±12.5	76.9±12.6	76.8±12.6
Fat mass (kg)	15.9±6.9	15.9±7.7	16.8±7.9
Bone Mineral Density			
Right hip (g/cm^2^)	1.02±0.13	1.02±0.14	
Lumbar spine (g/cm2)	1.2±0.16	1.2±0.17	
HLA supertype			
HLA-A24 (n, %)	31 (21)	38 (25)	
HLA-B07 (n, %)	63 (63)	85 (72)	
HLA-B08 (n, %)	33 (22)	26 (17)	
HLA-DR1 (n, %)	83 (56)	80 (53)	
HLA-DR9 (n, %)	7 (5)	6 (4)	
HLA-DQ1 (n, %)	100 (68)	101 (66)	
HLA-DQ3 (n, %)	86 (58)	95 (62)	

Results are expressed as mean ± standard deviation or %.

Abbreviations: ABC-3TC, abacavir-lamivudine; HLA, human leukocyte antigen; TDF-FTC, tenofovir-emtricitabine; NRTI, Nucleoside Reverse Transcriptase Inhibitor.

### Bone mineral density at baseline by HLA supertype

Participants carrying alleles belonging to DPw2, a class II supertype, had significantly lower spine BMD at baseline compared with the group without this supertype (1.161 vs. 1.203 g/cm^2^; mean difference −0.042; 95% confidence interval [CI] −0.083 to −0.001; p = 0.046). No significant differences were found for hip BMD at baseline for any supertypes groups. There was no difference in baseline BMD for participants expressing DQ3 compared with the ones not carrying DQ3 supertype (hip: 1.022 vs. 1.023, diff −0.001, 95%CI −0.034 to 0.030, p = 0.903; spine: 1.188 vs. 1.196, diff: 0.008, 95% −0.047 to 0.029, p = 0.656).

We explored the association between baseline BMD and HLA supertypes while considering the NRTI type participants were taking at baseline (TDF vs. non-TDF). There was a significant interaction between type of NRTI at baseline and HLA supertype for B07 (p-interaction  =  0.005) and B27 (p-interaction  =  0.014) on hip BMD. On spine BMD at baseline, HLA-A03 (p-interaction  =  0.009) and HLA-B58 (p-interaction  =  0.08) had an interaction with type of baseline NRTI.

Participants that were on TDF at study entry and were carrying A03 or DPw2 had significantly lower spine BMD at baseline than those on TDF without these supertypes (1.127 vs. 1.200 g/cm^2^; diff −0.073; 95%CI −0.137 to −0.008; p = 0.029 and 1.112 vs. 1.187 g/cm^2^; diff −0.075; 95%CI −0.150 to −0.001; p = 0.049, respectively). For baseline hip BMD, carrying B07 was associated with low BMD (0.978 vs. 1.072 g/cm2; diff −0.094; 95%CI −0.150 to −0.038; p = 0.001) while B27 was associated with higher BMD (1.057 vs. 0.991 g/cm^2^; diff 0.066; 95% 0.001 to 0.131; p = 0.046). For the group that were on TDF-containing regimen at study entry, there was no difference in baseline BMD by DQ3 status (hip: 1.006 vs. 1.019, diff −0.013, 95%CI −0.065 to 0.040, p = 0.627; spine: 1.159 vs. 1.181, diff: −0.022, 95% −0.089 to 0.044, p = 0.501).

For the group that was naïve to both TDF and ABC at baseline, there were significant differences for participants carrying HLA-A03 (1.122 vs. 1.165 g/cm^2^; diff 0.056; 95% 0.004 to 0.108; p = 0.035) supertype for spine BMD at baseline compared to patients not expressing this supertype. For hip BMD there was a significant difference for B27 supertype (0.982 vs. 1.058 g/cm^2^; diff −0.076; 95%CI −0.134 to −0.018; p = 0.010).

### Change in bone mineral density over 96 weeks by HLA supertype

We explored the association between change in BMD over 96 weeks and HLA supertypes. Supertypes that belong to class II were mainly associated with change in hip BMD (Table 2). Less bone loss at both the hip and spine was seen between those expressing HLA DQ3 compared to no DQ3 (hip: 0.003 vs. −0.006 g/cm^2^; diff 0.009; 95%CI 0.002 to 0.017; p = 0.016; spine: 0.006 vs. −0.006 g/cm^2^; diff 0.012; 95%CI 0.001 to 0.023; p = 0.041).

**Table 2:** Hip and spine absolute BMD change over 96 weeks by HLA.

		BMD change over 96 wks (g/cm^2^)	
HLA supertype		Hip	Spine
Class I			
A02	no	−0.001 (n = 130)	−0.004 (n = 131)
	yes	−0.001 (n = 158)	0.005 (n = 158)
	P value	0.804	0.099
A24	no	−0.001 (n = 224)	0.005 (n = 224)
	yes	−0.002 (n = 64)	−0.013 (n = 65)
	P value	0.624	0.010
B07	no	−0.001 (n = 64)	−0.010 (n = 65)
	yes	0.001 (n = 140)	0.003 (n = 140)
	P value	0.867	0.067
Class II			
DR9	no	−0.001 (n = 274)	0.000 (n = 275)
	yes	0.015 (n = 13)	0.006 (n = 13)
	P value	0.076	0.664
DQ3	no	−0.006 (n = 111)	−0.006 (n = 111)
	yes	0.003 (n = 177)	0.006 (n = 178)
	P value	0.016	0.041

Results are expressed as means and only presented for supertypes where a p<0.1 was observed at either site.

### Change in bone mineral density over 96 weeks by HLA supertype and randomised arm

Similarly to the whole cohort, no class I allele were associated with hip BMD change when the results were stratified by randomisation (Table S1). While there was a trend towards less bone loss with DQ3 for participants randomised to TDF-FTC, this association did not reach statistical significance (−0.003 vs. −0.014 g/cm^2^; diff 0.011; 95% −0.001 to 0.024; p = 0.066). For change in spine BMD, participants that were carrying HLA-A24 supertypes and were randomized to TDF-FTC experienced more bone loss compared with no-A24 (−0.024 vs. −0.002 g/cm2; diff 0.022; 95% 0.003 to 0.04; p = 0.021). The opposite effect was found for HLA-B07 – Participants carrying HLA-B07 experienced less bone loss compared with no-B07 (−0.001 vs. −0.029 g/cm2; diff 0.028; 95% 0.007 to 0.050 to; p = 0.01). The proportions of the different HLA supertypes in each treatment arm were similar (<10% difference) for nearly all HLA supertypes.

Investigating the interaction between HLA supertype and randomisation on hip BMD change over 96 weeks, the supertypes with p-interaction<0.1 were: HLA-A03 (p-interaction  =  0.096), B44 (p-interaction  =  0.08) and DR3 (p-interaction  =  0.08). For change in spine BMD, B07 (p-interaction  =  0.069), B08 (p-interaction  =  0.053), and DR1 (p-interaction  =  0.064) had an interaction with randomisation.

**Figure 2:**
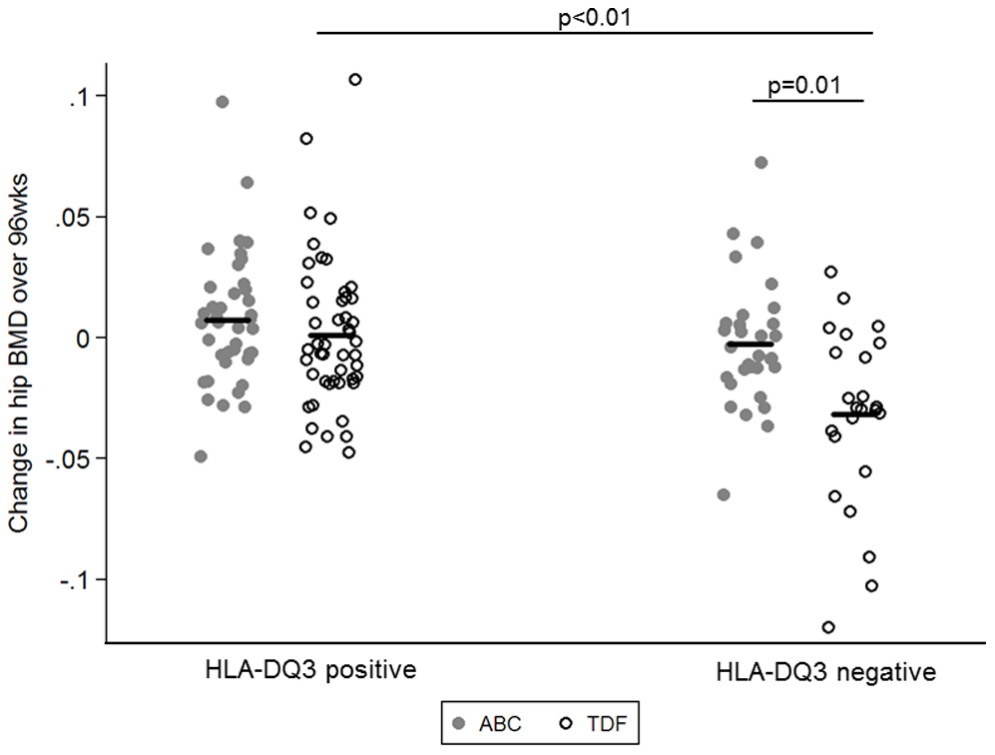
Absolute change over 96 weeks in hip BMD by HLA-DQ3 supertype and randomisation in participants naïve to both ABC and TDF at study entry.

**Figure 3:**
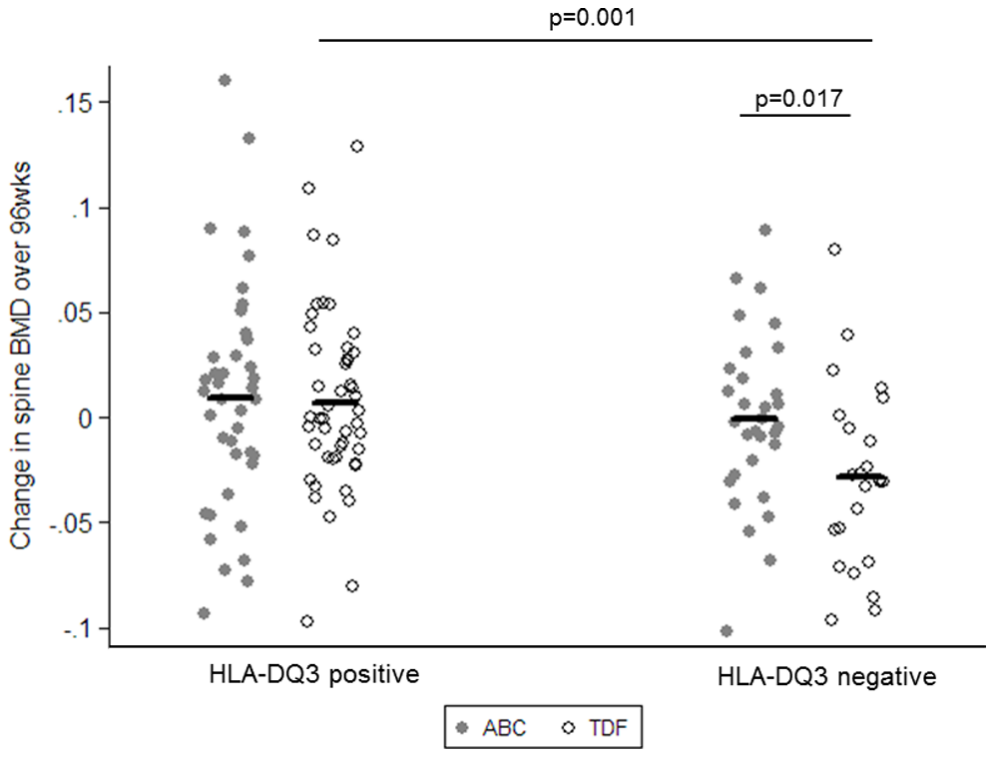
Absolute change over 96 weeks in spine BMD by HLA-DQ3 supertype and randomisation in participants naïve to both ABC and TDF at study entry.

In a sensitivity analysis of patients that were naïve to both ABC and TDF at baseline and randomised to TDF-FTC, DQ3 was significantly associated with less bone loss at both the hip (Figure 2) and spine (Figure 3), compared with those not carrying DQ3 (hip: 0.001 vs. −0.032 g/cm^2^; diff 0.033; 95%CI 0.017 to 0.049; p<0.001; spine: 0.007 vs. −0.023 g/cm^2^; diff 0.035; 95%CI 0.014 to 0.056; p = 0.001).

## Discussion

The relationship between HLA supertypes and bone mineral density has not been investigated in HIV infection. In this study, we show that different HLA supertypes may be linked to bone density status. At baseline, the class II supertype DPw2 was associated with lower spine BMD. Class II HLA supertypes were mainly associated with change in hip BMD over 96 weeks. Overall, individuals carrying alleles that belong to the HLA-DQ3 supertype had increased BMD at both the hip and the spine compared to the decrease seen in participants not carrying DQ3 supertype. A potential beneficial effect of carrying DQ3 on BMD was seen in participants naïve to both TDF and ABC at study entry that were randomised to TDF-FTC in an analysis stratified by randomisation group.

A study of postmenopausal women found an increased frequency of HLA-DR15 and -DQ6 supertypes in osteoporotic women compared to healthy controls [9]. The authors also report that a higher frequency of HLA-B07 was found in the osteoporotic group. This is in contrast to our finding that B07 was associated with less bone loss at the spine over 96 weeks. There are a few reasons that may explain the different results. First, the two studies have different populations, Greek women versus mainly Australian men in our cohort. Furthermore, the Greek study used BMD measured at the forearm which contains different components of cortical and trabecular bone than the hip and spine. It is possible that genetic variation in bone remodeling has different effects for cortical and trabecular bone. Genetic variation at different skeletal sites [22], may also support our finding that hip BMD change was associated with different supertypes than the spine. There are many factors that influence BMD; determining the effect of gene-environment interaction on BMD is particularly complicated if multiple genes are involved.

Bone is constantly remodeled by bone-resorbing osteoclasts and bone-forming osteoblasts, excess osteoclast activity can lead to bone disease [23]. There are complex regulatory circuits that are shared between the immune and the skeletal systems [1]. MHC class II transactivator (CIITA) is an immune regulator that has been referred to as the master control factor for MHC-II genes [24]. It was recently shown that CIITA also acts as a modulator of osteoclast function and provides negative feedback regulation to maintain bone homeostasis in vivo [25]. Furthermore, the MHC locus was reported to be associated with BMD and hip and forearm fractures in three populations of European descent [26], however, this relationship was not found in a recent meta-analysis of 17 genome-wide association studies [27].

Tsuji et al. previously suggested that the association between HLA and BMD can be explained by an interaction between an HLA-haplotype and tumor necrosis factor (TNF) gene, as both are located near each other on the same chromosome 6p [7]. The TNF gene cluster and other immune regulatory genes are contained within the HLA locus, a region of great polymorphic variation, and there is often linkage disequilibrium with the HLA genes and with each other [28]. TNF is a cytokine that stimulates bone resorption and inhibits bone formation [29]. Individual differences in TNF secretion due to genetic differences could have an effect on BMD that may be attributed to HLA haplotypes.

The MHC plays an important role in some adverse drug reactions. In HIV, a genetic susceptibility to abacavir hypersensitivity was found in individuals carrying the HLA-B*5701, HLA-DR7, and HLA-DQ3 haplotype [13]. HLA-DQ3 was found in our study to have a beneficial effect on BMD, at both the hip and the spine. The same effect was found when the results were stratified for the participants that were naïve to both ABC and TDF at baseline and were randomised to TDF-FTC, possibly suggesting the individuals carrying HLA-DQ3 may be resistant to TDF-induced bone loss, however, due to the small size of this sub-group these results should be interpreted with caution. A positive effect of HLA supertypes on peripheral fat-loss induced by thymidine-analogue NtRTI was also shown in a recent study of HIV-infected adults [17].

The enrolment criteria for the STEAL study excluded patients carrying HLA-B*5701. There is an extended haplotype that includes HLA-B*5701, HLA-DR7, and HLA-DQ3 [13]. The frequency of this haplotype in an Australian cohort of HIV-negative bone-marrow donors (n = 3212) was 0.1% [13]. Out of the 441 individuals assessed for eligibility for the STEAL study, 26 were excluded due to HLA-B*5701 positivity [19]. Applying the probability described (0.1%), our exclusion of HLA-B*5701 may have resulted in exclusion of approximately one individual carrying DQ3 from our cohort, which is unlikely to significantly bias our results.

Our study is limited by the 2-digit genotyping and by the fact that some of the alleles could not be assigned into supertypes. However, our cohort is ethnically homogenous allowing a more accurate categorization of the alleles into their supertypes. Our statistical power was limited due to the small size of our cohort; in addition, the absolute BMD differences over 96 weeks were small. This may mean that smaller effect sizes were not detected. Furthermore, the effect values found for different HLA stratification are very small and not clinically meaningful, including the means differences in BMD change by DQ3 status. Therefore the results should be interpreted with the caution and the significant p-values should only be used as indicators of association. Another limitation is that we tested the association at the supertype level. If two alleles in the same supertype group had different effects on BMD, their influence may have been masked.

In conclusion, in this cohort of HIV-infected adults, there was an association between bone status and HLA supertypes, particularly HLA-DQ3. Although these results are not clinically useful in the prediction of bone loss risk at present, further exploration of the role of HLA alleles and other candidate genes may provide useful insight into the complex relationship between bone and HIV. Larger scale studies in HIV positive patient cohorts, with more detailed sequencing may be required.

## Supporting Information

Checklist S1 **CONSORT Checklist.**
(PDF)

Protocol S1 **Trial Protocol.**
(PDF)

Table S1 **Absolute change over 96 weeks in (a) hip and (b) spine bone mineral density by HLA stratified by randomisation.** Note: Results are expressed as means and only presented for supertypes where a p<0.1 was observed at either strata.
(DOCX)
