# Supplementary material for: HLA Alleles Association with Changes in Bone Mineral Density in HIV-1-Infected Adults Changing Treatment to Tenofovir-Emtricitabine or Abacavir-Lamivudine
Source: PLoS One. 2014 Mar 28;9(3):e93333. doi: 10.1371/journal.pone.0093333 (PMC3969319; doi:10.1371/journal.pone.0093333)
Supplement: Table S1 — Absolute change over 96 weeks in (a) hip and (b) spine bone mineral density by HLA stratified by randomisation. Note: Results are expressed as means and only presented for supertypes where a p<0.1 was observed at either strata. (DOCX) [file pone.0093333.s003.docx]

## Web table S3a: Absolute change over 96 weeks in hip bone mineral density by HLA stratified by randomisation

|  |  | **Wk96 change Hip BMD** | |  |
| --- | --- | --- | --- | --- |
| **HLA supertype** |  | **ABC-3TC** | **TDF-FTC** | **P value** |
| **Class II** |  |  |  |  |
| DQ3 | no | 0.001 (n=56) | -0.014 (n=55) | **0.016** |
|  | yes | 0.009 (n=85) | -0.003 (n=92) | **0.010** |
|  | P value | 0.069 | 0.066 |  |
| DPw4 | no | 0.012 (n=33) | -0.004 (n=41) | **0.024** |
|  | yes | 0.002 (n=96) | -0.006 (n=92) | 0.077 |
|  | P value | **0.044** | 0.766 |  |

**Note.** Results are expressed as means and only presented for supertypes where a p<0.1 was observed at either strata

## Web table S3b: Absolute change over 96 weeks in spine bone mineral density by HLA stratified by randomisation

|  |  | **Wk96 change Spine BMD** | |  |
| --- | --- | --- | --- | --- |
| **HLA supertype** |  | **ABC-3TC** | **TDF-FTC** | **P value** |
| **Class I** |  |  |  |  |
| A03 | no | 0.014 (n=87) | -0.008 (n=79) | **0.003** |
|  | yes | 0.001 (n=54) | -0.006 (n=69) | 0.453 |
|  | P value | 0.096 | 0.827 |  |
| A24 | no | 0.011 (n=111) | -0.002 (n=113) | **0.036** |
|  | yes | 0.001 (n=30) | -0.024 (n=35) | 0.061 |
|  | P value | 0.242 | **0.021** |  |
| B07 | no | 0.006 (n=34) | -0.029 (n=31) | **0.005** |
|  | yes | 0.008 (n=60) | -0.001 (n=80) | 0.331 |
|  | P value | 0.868 | **0.01** |  |
|  | unassigned | 0.013 (n=48) | -0.002 (n=37) | 0.109 |
| B08 | no | 0.005 (n=111) | -0.006 (n=123) | 0.098 |
|  | yes | 0.025 (n=30) | -0.013 (n=25) | 0.003 |
|  | P value | **0.031** | 0.482 |  |
| **Class II** |  |  |  |  |
| DR1 | no | 0.019 (n=63) | -0.001 (n=70) | **<0.001** |
|  | yes | 0.002 (n=78) | -0.005 (n=77) | 0.413 |
|  | P value | **0.025** | 0.647 |  |
| DQ3 | no | 0.002 (n=56) | -0.015 (n=55) | 0.051 |
|  | yes | 0.014 (n=85) | -0.002 (n=93) | **0.032** |
|  | P value | 0.127 | 0.131 |  |

**Note.** Results are expressed as means and only presented for supertypes where a p<0.1 was observed at either strata
